# Presentation of the septic patient to the emergency department with respect to age and sex – a retrospective cross-sectional study

**DOI:** 10.1186/s12873-022-00759-6

**Published:** 2022-12-14

**Authors:** Eric A. Larsson, Ulrika M. Wallgren, Anna Su, Jennifer Short, Lisa Kurland

**Affiliations:** 1 Department of Medical Sciences, Örebro University, Campus USÖ, Södra Grev Rosengatan 32, 701 12 Örebro, Sweden; 2 Centre of Clinical Research and Education, Region Värmland, Älvgatan 49, 651 85 Karlstad, Sweden; 3 Department of Clinical Science and Education, Karolinska Institutet, Södersjukhuset, Sjukhusbacken 10, 118 83 Stockholm, Sweden; 4 Fisksätra Vårdcentral (Primary Health Care Center), Fisksätra Torg 20, 133 41 Saltsjöbaden, Sweden; 5 Intern Physician, Sankt Göran Hospital, Sankt Göransplan 1, 112 19 Stockholm, Sweden; 6 Department of Urology, Linköping University Hospital, Universitetssjukhuset, 581 85 Linköping, Sweden; 7 Department of Emergency Medicine, Örebro University Hospital, Södra Grev Rosengatan 18, 703 62 Örebro, Sweden

**Keywords:** Emergency Department, Sepsis, Symptoms, Age, Sex

## Abstract

**Objective:**

It is challenging to identify sepsis in the emergency department, in part due to the non-specific presentation of septic patients. Current clinical sepsis screening tools rely on vital signs but many patients present with near normal vital signs and are therefore not identified as septic. This suggests that variables, e.g. signs and symptoms, need to be included to improve sepsis detection in the emergency department. Our hypothesis was that the presentation of sepsis differs based age and sex. The potential differences in presentation could be used to apply to future sepsis screening tools. The aim was to analyze the prevalence of keywords reflecting the presentation of septic patients in the emergency department in relation to age and sex.

**Method:**

Retrospective cross-sectional study. Keywords reflecting sepsis presentation to the emergency department were quantified and compared between age categories and the sex. 479 patients admitted to the emergency department of Södersjukhuset, Stockholm during 2013 and discharged with an ICD-10 code consistent with sepsis were included. We adjusted for multiple comparisons by applying Bonferroni-adjusted significance levels for all comparisons.

**Result:**

“Pain” and “risk factors for sepsis” were significantly more common among patients younger than 65 years as compared with those 75 years and older: (*n* = 87/137; 63.5% vs *n* = 99/240; 41.3%, *P*-value < 0.000) and (*n* = 74/137; 54.0% vs 55/240; 22.9%, *P*-value < 0.000) respectively. “Risk factors for sepsis” was also significantly more common among patients between 65 and 74 years as compared with those 75 years and older: (*n* = 43/102; 42.2% vs 55/240; 22.9%, *P*-value < 0.000). “Pain” and “gastrointestinal symptoms” were significantly more common among women as compared with men: (*n* = 128/224; 57.1% vs *n* = 102/255; 40.0%, *P*-value < 0.000) and (*n* = 82/244; 36.6% vs *n* = 55/255; 21.6%, *P*-value < 0.000) respectively.

**Conclusion:**

The keywords “pain” and “risk factors for sepsis” were more common among younger patients and “pain” and “gastrointestinal symptoms” were more common among women. However, most keywords had a similar prevalence irrespective of age and sex. The results could potentially be used to augment sepsis screening tools or clinical decision tools.

**Supplementary Information:**

The online version contains supplementary material available at 10.1186/s12873-022-00759-6.

## Introduction

Sepsis is a common and life-threatening medical emergency and immediate medical attention is needed [1]. Nevertheless, health care personnel often fail to identify septic patients [2]. This may be explained, in part, by the non-specific presentation of sepsis [3–5]. Most of the sepsis screening tools in use today are based on vital signs, which is a limitation in the identification of the septic patient as approximately one third of the patients with serious infections do not present with fever and one third present with near normal vital signs [6, 7]. Hence, additional information, e.g., that of symptom presentation, can be assumed to be needed.

Identification of sepsis among older people is especially challenging as older patients often have comorbidities which may mask the underlying infection and sepsis [8]. Moreover, non-specific presentations of sepsis are more common among older people [8, 9]. There are also no prior studies of the effect of sex on symptom presentation in septic patients. Prior literature focuses mainly on pathophysiological mechanisms and differences in outcome between men and women while knowledge of symptom presentation in relation to sex is limited [10, 11].

Hence, we hypothesize that a better understanding of the presentation of the septic patient in relation to age and sex could add to the identification of the septic patient in the ED. Therefore, the aim of the current study was to analyze the prevalence of keywords describing the presentation of septic patients to the ED with respect to age and sex.

## Methods

### Study design

The current study is a retrospective, cross-sectional study. The prevalence of the 90 keywords, identified in a prior study [12] using a sequential mixed methods approach [13–15], were quantified among a cohort of adult patients presenting to the ED and discharged with an ICD-code compatible with sepsis. The patients were stratified with respect to age and sex. The overall study design used in the current study had a similar approach used in a previous study where sepsis presentation based on mode of arrival to the ED was explored [16].

### Study setting

The study setting was the ED of Södersjukhuset, Stockholm, Sweden. Södersjukhuset was at the time the largest ED in all of the Nordic countries with 110,000 -120,000 ED visits annually [17].

### Study population

#### Inclusion criteria

Patients ≥ 18 years of age and admitted to in-hospital care at Södersjukhuset via the ED between January 1^st^ 2013 and December 31^st^ 2013 and subsequently discharged with an ICD-10-code (International Classification of Disease) consistent with sepsis (A02.1, A22.7, A26.7, A32.7, A39.2, A39.4, A40.0 – A40.3, A48—A49, A41.0—A41.5, A41.8—A41.9, A42.7, B37.7, R57.2, R65.0–65.1) were included.

#### Exclusion criteria

Exclusion criteria were 1) interfacility transports of patients already treated for sepsis, 2) lack of ED records, 3) patients with healthcare-associated infections (HCAI), defined as a sepsis-onset ≥ 48 h after admission to in-hospital care. HCAI was determined by chart review of in-hospital records for all patients admitted to in-hospital care without signs or symptoms of infection or sepsis in the ED, but later developed signs and symptoms of infection or sepsis > 48 h after admission to in-hospital care and were subsequently discharged with an ICD-10 code compatible with sepsis.

### Definitions

The current study used the term “keywords” as a hypernym for signs, symptoms and other factors describing the presentation of the septic patient to the ED. Examples of primary keywords are “abdominal pain”, “back pain”, “extremity pain” and “vomiting”, “diarrhea”, “obstipation” which together formed the combined keywords “pain” and “gastrointestinal symptoms” respectively.

### Data collection and analysis

90 primary and 14 combined keywords in total, were quantified. Quantification of keywords was done in the following sections of the ED records documented by the admitting physician (IT based and documented after dictation): “chief complaint” (reflecting the patient’s own wording upon ED arrival), “current medical history” (reflecting onset of current symptomology) and “preliminary assessment” (final part of the ED record including assessment of reason for the patient to attend the ED including a plan forward). Symptoms with an onset within 3 weeks preceding the index ED visit were defined as “new” and considered relevant. Chart review of free text to assign keywords to patient cases was performed manually by two abstractors (AS and JS) under the supervision of (UW) who had participated in the prior study where the keywords were derived [12]. Disagreements regarding keyword categorization was discussed between the two abstractors and the supervising researcher until agreement was achieved.

Patients were stratified based on age and sex. Three age categories were created: < 65, 65–74 and ≥ 75 years of age based on cut-off values from Angus et al. [18]. Only keywords with a prevalence of 20% or more were presented in the manuscript. The keywords in their entirety were presented in supplemental tables.

### Statistics

SPSS® (Statistical Package for Social Sciences SPSS®, IBM®, student version 22.0) was used for statistical analysis. The analysis of categorical values was performed using Chi-Square tests. Fisher’s exact test was used when expected cell count was < 5. *P*-values are presented in the tables without adjustment for multiple comparisons. The Bonferroni-adjusted significance level is presented in the footnote of each table, calculated by dividing the significance level 0.05 with the number of performed comparisons. Only two tailed *P*-values that remained significant according to the Bonferroni-adjusted significance level were considered significant in the current study.

### Ethical approval

Stockholm Regional Ethical Review Board approval was obtained for this study (reference number 2012/1288–31/3). The study was performed according to the ethical standards of the 1964 Declaration of Helsinki and its later amendments. However, no written informed consent was obtained from the study participants as this was a register-based study and thus not needed according to Swedish practice [19].

## Results

### Characteristics

**Fig. 1:**
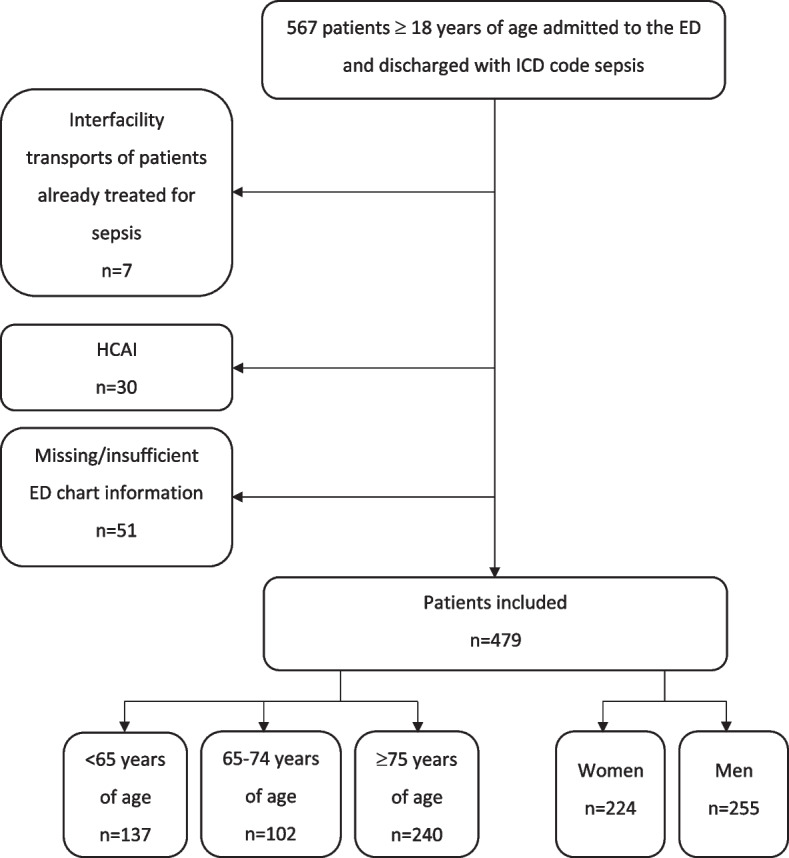
Flow chart illustrating patient inclusion and exclusion. Flow chart for inclusion and exclusion of adult patients arriving to the ED of Södersjukhuset during 2013 and discharges with an ICD code compatible with sepsis. ED = Emergency Department, ICD = International Classification of Disease, HCAI = Health Care Associated Infection

A total of 479 patients were included, see Fig. 1 for flow chart of inclusion and exclusion of patients. The median age was 75 years of age (IQR 61–85). 224 (46,8%) patients were women. 137 (28.6% were < 65 years of age, 102 (21.3%) were 65–74 years of age and 240 (50.1%) were ≥ 75 years of age. 99 (20.7%) patients died during in-hospital care. See Table 1 for patient characteristics.

**Table 1:** Demographic data of patients with sepsis admitted to the ED of Södersjukhuset in 2013

	**479 patients with sepsis admitted during 2013**	
	**Median (IQR)**	
**Age, years**	75 (61–85)	
**Age categories:**	**Number (%)**	**Women (%)**
- < 65 years	137/479 (28.6)	73/137 (53.3)
- 65–74 years	102/479 (21.3)	35/102 (34.3)
- ≥ 75 years	240/479 (50.1)	116/240 (48.3)
**Sex:**	**Number (%)**	**Age median (IQR)**
- Women	224/479 (46.8)	76 (56–86)
- Men	255/479 (53.2)	73 (64–84)
**In-hospital mortality:**	**Number (%)**	
- Total group	99/479 (20.7)	
- < 65 years	9/137 (6.6)	
- 65–74 years	15/102 (14.7)	
- ≥ 75 years	75/240 (31.3)	
- Women	52/224 (23.2)	
-Men	47/255 (18.4)	

Patient characteristics of the entire group of patients admitted to the ED of Södersjukhuset during 2013 and discharged with an ICD-10 code compatible with sepsis

*ED* Emergency department, *IQR* Interquartile range

#### Keywords with a prevalence exceeding 20% in the entire group of ED patients

Twelve keywords had a prevalence of ≥ 20% in the entire cohort (*n* = 479): “abnormal, or suspected abnormal temperature” (*n* = 319, 66.6%), “pain” (*n* = 230, 48.0%), “abnormal breathing” (*n* = 210, 43.8%), “risk factors for sepsis” (*n* = 172, 36.0%), “abnormal circulation” (*n* = 163, 34.0%), “temporal deterioration” (*n* = 144, 30.1%), “gastrointestinal symptoms” (*n* = 137, 28.6%), “acute altered mental status” (*n* = 127, 26.5%), “abnormal skin” (*n* = 125, 26.1%), “abnormal urination” (*n* = 118, 24.6%), “loss of energy” (*n* = 113, 23.6%) and “decreased mobility” (*n* = 106, 22.1%), see Tables 2 and 3.

**Table 2:** Prevalence of keywords* exceeding 20% among ED patients discharged with ICD-10 code sepsis based on age

		**Prevalence**								
		**Entire group of ED patients** **(*n* = 479)**		** < 65 years** **(*n* = 137)**		**65–74 years** **(n = 102)**		** ≥ 75 years** **(n = 240)**		
**Order**	Keyword*	**Number of patients**	**Percent (%) and 95% CI**	**Number of patients**	**Percent (%) and 95% CI**	**Number of patients**	**Percent (%) and 95% CI**	**Number of patients**	**Percent (%) and 95% CI**	*P*-value**
**1**	**Abnormal, or suspected abnormal temperature** In turn including primary keywords shivering OR hypothermia OR the following combined keywords: Confirmed or suspected fever, Confirmed abnormal temperature (confirmed fever or hypothermia)	319	66.6 (62.3–70.7)	97	70.8 (62.4–78.3)	72	70.6 (60.7–79.2)	150	62.5 (56.0–68.6)	0.163
**2**	**Pain** Abdominal/extremity/back/ undefined/urinary tract/joint/ chest/general/headache/throat/ wound/painful muscle cramp/ positive Pasternatsy´s sign (costovertebral angle tenderness)	230	48.0 (43.6–52.5)	87	63.5 (54.9–71.6)	44	43.1 (33.4–53.3)	99	41.3 (35.0–47.8)	**0.000094** < 65 years vs 65–74 years: 0.002 < 65 years vs ≥ 75 years: **0.000032** 65–74 years vs ≥ 75 years: 0.746
**3**	**Abnormal breathing** Tachypnea, low oxygen saturation, airway secretions, breathing difficulties, cough, or obstructive breathing	210	43.8 (39.5–48.3)	44	32.1 (24.4–40.6)	51	50.0 (39.9–60.1)	115	47.9 (41.4–54.4)	0.004 < 65 years vs 65–74 years: 0.005 < 65 years vs ≥ 75 years: 0.003 65–74 years vs ≥ 75 years: 0.724
**4**	**Risk factors for sepsis** Known ongoing or recent infection, current antibiotic treatment, recent invasive procedures, substance abuse, compromised immune system, chronically compromised breathing	172	36.0 (31.7–40.3)	74	54.0 (45.3–62.6)	43	42.2 (32.4–52.3)	55	22.9 (17.8–28.8)	**3.6677E-9** < 65 years vs 65–74 years: 0.070 < 65 years vs ≥ 75 years: **9.2784E-10** 65–74 years vs ≥ 75 years: **0.000318**
**5**	**Abnormal circulation** Weak pulse or difficulties to palpate the pulse, peripheral coldness, cardiac arrest, tachycardia, low blood pressure, prolonged capillary refill time or non-measurable circulatory variables	163	34.0 (29.9–38.4)	47	34.3 (26.4–42.9)	40	39.2 (29.7–49.4)	76	31.7 (25.8–38.0)	0.402
**6**	**Temporal deterioration** Stated deterioration or expressions describing a temporal change	144	30.1 (26.1–34.3)	43	31.4 (23.7–39.9)	34	33.3 (24.3–43.4)	67	27.9 (22.3–34.1)	0.560
**7**	**Gastrointestinal symptoms** Vomiting, diarrhoea, reduced amount of stool, gastrointestinal bleeding, obstipation, pale faeces	137	28.6 (24.7–32.8)	44	32.1 (24.4–40.6)	27	26.5 (18.2–36.1)	66	27.5 (22.0–33.6)	0.549
**8**	**Acute altered mental status** Abnormal behaviour or level of consciousness (excluding previously known dementia or mental retardation without statement worse) OR abnormal verbal response defined as no/decreased verbal response	127	26.5 (22.8–30.6)	28	20.4 (14.0–28.2)	22	21.6 (14.0–30.8)	77	32.1 (26.2–38.4)	0.021 < 65 years vs 65–74 years: 0.832 < 65 years vs ≥ 75 years: 0.015 65–74 years vs ≥ 75 years: 0.050
**9**	**Abnormal skin** Paleness, wounds or wound infection, sweaty, cyanosis, redness, icterus, mottling, bruises, rash, blisters or peteckiae, change of skin turgor, exuding skin	125	26.1 (22.4–30.2)	46	33.6 (25.7–42.1)	20	19.6 (12.4–28.6)	59	24.6 (19.3–30.5)	0.039 < 65 years vs 65–74 years: 0.017 < 65 years vs ≥ 75 years: 0.061 65–74 years vs ≥ 75 years: 0.318
**10**	**Abnormal urination** Abnormal urination (such as haematuria without trauma, bad smelling or cloudy urine, increased frequency of urination) OR urinary tract pain OR decreased urinary volumes OR dysfunction of urinary catheters defined as obstruction/leakage/problematic urinary catheters including nefrostomias	118	24.6 (21.0–28.7)	23	16.8 (11.0–24.1)	28	27.5 (19.1–37.2)	67	27.9 (22.3–34.1)	0.041 < 65 years vs 65–74 years: 0.047 < 65 years vs ≥ 75 years: 0.015 65–74 years vs ≥ 75 years: 0.930
**11**	**Loss of energy** Defined as fatigue, weakness, faintness or similar expressions	113	23.6 (20.0–27.6)	28	20.4 (14.0–28.2)	19	18.6 (11.6–27.6)	66	27.5 (22.0–33.6)	0.123
**12**	**Decreased mobility** in turn including primary keywords remained sitting or lying in an abnormal way OR decreased miscellaneous mobility OR the following combined keywords: “weakness of the legs” and” fallen or being found on the floor”	106	22.1 (18.6–26.1)	18	13.1 (8.0–20.0)	22	21.6 (14.0–30.8)	66	27.5 (22.0–33.6)	0.005 < 65 years vs 65–74 years: 0.084 < 65 years vs ≥ 75 years: 0.001 65–74 years vs ≥ 75 years: 0.251

*ED* Emergency Department, *CI* Confidence Interval

^*^The prevalence of all keywords (both primary and combined) exceeding 20% in the entire group of patients admitted to the ED of Södersjukhuset during 2013 and discharged with an ICD-10 code compatible with sepsis. The table illustrates the prevalence in the entire group and the prevalence based on age group

^**^for comparison between the three age groups using chi-square to identify any statistically significant difference. If statistical significance was identified in the first analysis a pairwise comparison using chi-square was then performed to identify between which age groups the statistical significance applied to. *P*-values are presented without adjustment for multiple comparisons. In total 3 × 12 = 36 tests were performed. Bonferroni-adjusted significance level is 0,05/36 = 0,0014. *P*-values indicating significant differences after adjustment for multiple comparisons by Bonferroni correction are bolded and considered significant in the current study

**Table 3:** Prevalence of keywords* exceeding 20% among ED patients discharged with ICD-10 code sepsis based on sex

		**Prevalence**						
		**Entire group of ED patients** **(*n* = 479)**		**Women** **(*n* = 224)**		**Men** **(*n* = 255)**		
**Order**	Keyword*	**Number of patients**	**Percent (%) and 95% CI**	**Number of patients**	**Percent (%)**	**Number of patients**	**Percent (%)**	*P*-value**
**1**	**Abnormal, or suspected abnormal temperature** In turn including primary keywords shivering OR hypothermia OR the following combined keywords: Confirmed or suspected fever, Confirmed abnormal temperature (confirmed fever or hypothermia)	319	66.6 (62.3–70.7)	155	69.2 (62.7–75.2)	164	64.3 (58.1–70.2)	0.258
**2**	**Pain** Abdominal/extremity/back/ undefined/urinary tract/joint/ chest/general/headache/throat/ wound/painful muscle cramp/ positive Pasternatsy´s sign (costovertebral angle tenderness)	230	48.0 (43.6–52.5)	128	57.1 (50.4–63.7)	102	40.0 (33.9–46.3)	**0.000179**
**3**	**Abnormal breathing** Tachypnea, low oxygen saturation, airway secretions, breathing difficulties, cough, or obstructive breathing	210	43.8 (39.5–48.3)	104	46.4 (39.8–53.2)	106	41.6 (35.5–47.9)	0.285
**4**	**Risk factors for sepsis** Known ongoing or recent infection, current antibiotic treatment, recent invasive procedures, substance abuse, compromised immune system, chronically compromised breathing	172	36.0 (31.7–40.3)	72	32.1 (26.1–38.7)	100	39.2 (33.2–45.5)	0.107
**5**	**Abnormal circulation** Weak pulse or difficulties to palpate the pulse, peripheral coldness, cardiac arrest, tachycardia, low blood pressure, prolonged capillary refill time or non-measurable circulatory variables	163	34.0 (29.9–38.4)	83	37.1 (30.7–43.7)	80	31.4 (25.7–37.5)	0.190
**6**	**Temporal deterioration** Stated deterioration or expressions describing a temporal change	144	30.1 (26.1–34.3)	69	30.8 (24.8–37.3)	75	29.4 (23.9–35.4)	0.740
**7**	**Gastrointestinal symptoms** Vomiting, diarrhoea, reduced amount of stool, gastrointestinal bleeding, obstipation, pale faeces	137	28.6 (24.7–32.8)	82	36.6 (30.3–43.3)	55	21.6 (16.7–27.1)	**0.000279**
**8**	**Acute altered mental status** Abnormal behaviour or level of consciousness (excluding previously known dementia or mental retardation without statement worse) OR abnormal verbal response defined as no/decreased verbal response	127	26.5 (22.8–30.6)	58	25.9 (20.3–32.1)	69	27.1 (21.7–33.0)	0.773
**9**	**Abnormal skin** Paleness, wounds or wound infection, sweaty, cyanosis, redness, icterus, mottling, bruises, rash, blisters or peteckiae, change of skin turgor, exuding skin	125	26.1 (22.4–30.2)	59	26.3 (20.7–32.6)	66	25.9 (20.6–31.7)	0.910
**10**	**Abnormal urination** Abnormal urination (such as haematuria without trauma, bad smelling or cloudy urine, increased frequency of urination) OR urinary tract pain OR decreased urinary volumes OR dysfunction of urinary catheters defined as obstruction/leakage/problematic urinary catheters including nefrostomias	118	24.6 (21.0–28.7)	46	20.5 (15.4–26.4)	72	28.2 (22.8–34.2)	0.051
**11**	**Loss of energy** Defined as fatigue, weakness, faintness or similar expressions	113	23.6 (20.0–27.6)	59	26.3 (20.7–32.6)	54	21.2 (16.3–26.7)	0.184
**12**	**Decreased mobility** in turn including primary keywords remained sitting or lying in an abnormal way OR decreased miscellaneous mobility OR the following combined keywords: “weakness of the legs” and” fallen or being found on the floor”	106	22.1 (18.6–26.1)	41	18.3 (13.5–24.0)	65	25.5 (20.3–31.3)	0.059

*ED* Emergency Department, *CI* Confidence Interval

^*^The prevalence of all keywords (both primary and combined) exceeding 20% in the entire group of patients admitted to the ED of Södersjukhuset during 2013 and discharged with an ICD-10 code compatible with sepsis. The table illustrates the prevalence in the entire group and the prevalence based on sex

^**^for comparison between sexes using Chi-Square analysis. *P*-values are presented without adjustment for multiple comparisons. In total 2 × 12 = 24 tests were performed. Bonferroni-adjusted significance level is 0,05/24 = 0,0021. *P*-values indicating significant differences after adjustment for multiple comparisons by Bonferroni correction are bolded and considered significant in the current study

99.6% of all septic patients had ≥ 1 of these twelve keywords, 96.9% had ≥ 2, 83.7% had ≥ 3 and 64.1% had ≥ 4 keywords.

#### Comparisons between age categories

For prevalence of all keywords, based on age, see Supplementary Table 1 (primary keywords) and Supplementary Table 2 (combined keywords).

“Pain” and “risk factors for sepsis” were significantly more common among patients < 65 years of age as compared with those ≥ 75 years of age: (*n* = 87/137; 63.5% vs *n* = 99/240; 41.3%, *P*-value < 0.000) and (*n* = 74/137; 54.0% vs 55/240; 22.9%, *P*-value < 0.000) respectively. “Risk factors for sepsis” was also significantly more common among patients 65–74 years of age as compared with those ≥ 75 years of age: (*n* = 43/102; 42.2% vs 55/240; 22.9%, *P*-value < 0.000), see Table 2.

#### Comparisons between the sexes

For prevalence of all keywords, based on sex, see Supplementary Table 3 (primary keywords) and Supplementary Table 4 (combined keywords).

“Pain” and “gastrointestinal symptoms” were significantly more common among women as compared with men: (*n* = 128/224; 57.1% vs n = 102/255; 40.0%, *P*-value < 0.000) and (*n* = 82/244; 36.6% vs *n* = 55/255; 21.6%, *P*-value < 0.000) respectively, see Table 3.

## Discussion

The keywords “pain” and “risk factors for sepsis” were more common among younger patients and “pain” and “gastrointestinal symptoms” were more common among women. However, most keywords had a similar prevalence irrespective of age and sex.

### Presentation in relation to age

Pain was more common among septic patients below the age of 65 as compared to those ≥ 75 years of age. Prior literature indicates that age does not affect pain tolerance per se, but that aging may decrease the sensitivity for some types of pain [20]. Also, older patients have, a greater prevalence of chronic conditions, e.g. dementia and post stroke, that might affect the ability to communicate symptoms [21]. Hence, we speculate that the older septic patients may have difficulties in expressing pain, as compared with the younger patients.

Patients below the age of 75 had a higher prevalence of the keyword “risk factors for sepsis”. The combined keyword “risk factors for sepsis” includes “external” risk factors for sepsis such as recent invasive procedures, substance abuse and ongoing antibiotic treatment. Older people are predisposed to infections due to a general decline in their physiological, anatomical and immunological defense against microbes [22], a.k.a. immunosenescence, which is likely to contribute to the overall higher prevalence of sepsis among older people [9]. We speculate that younger people may, so to speak, require an external risk factor for sepsis, which is not observed among older people. Approximately half of the patients included in the current study were above 75 years of age and had an in-hospital mortality of nearly one in three, which was substantially higher than that of the other age groups.

### Presentation in relation to sex

Women had a higher prevalence of the keywords “pain” and “gastrointestinal symptoms”. The keyword “pain” involves all types of pain, without specific focus. However, abdominal pain was the most common type of pain. Previous literature indicates that women as a group may experience pain more often [23]. Also, previous literature indicate that women generally report more intense and more frequent bodily symptoms, while men have been shown to be less prone to admit feeling ill or experiencing pain [24]. The findings in the current study are in line with other literature that has investigated sex-related differences in the perception of pain in other acute medical conditions, which points to the need to include this information when assessing patient in the ED [25–27].

Women more frequently reported gastrointestinal symptoms which is supported by prior studies [28, 29]. Estrogen has previously been connected to nausea, vomiting and reduced appetite [30] and we speculate that it may play a role also in sepsis. However, the exact mechanisms explaining the difference in prevalence between the sexes remains unclear.

No keyword with a prevalence exceeding 20% was more common among men than women. It is possible that men underreport the presence of symptoms as compared to women (as discussed above), which may affect the current results.

### Strengths and limitations

This is the first study, to our knowledge, to analyze differences in the presentation of sepsis to the ED with respect to age and sex. The understanding of the presentation including the symptomatology of sepsis in the ED may lead to an increased identification and enable timely treatment of the septic patient.

There are several limitations to the current study. The retrospective study design has inherent limitations e.g. missing data. Only predefined sections of the ED medical records were analyzed for keywords and are written by the admitting doctor. Due to interindividual variations in the competence and experience of the doctors working in the ED as well as a variations in work load the detail of the records may vary. Also, factors such as baseline co-morbidities, laboratory results and information as to which ward the patients were admitted to (e.g. ICU) were not retrieved at the time of data extraction which is a limitation of the current study. However, this was not the aim of the study.

Multiple comparisons were performed. Bonferroni corrections were therefore applied to adjust the levels of significance. However, the Bonferroni correction is overly strict and may infer a type II error, which may have resulted in true differences being regarded as non-significant.

Also, the generalizability of the results may be limited since this is a single center study. However, the ED of Södersjukhuset was at the time the largest in Scandinavia. Hence it is likely that the results are generalizable to other ED settings.

The inclusion criteria in current study were based on ICD-10 codes consistent with sepsis and ICD-10 coding is a crude method for identification of the septic patient, however, the only method applicable for registry studies.

Assignment of keywords is inherently in part subjective which can be viewed as a limitation. However, assignment of keywords as in the current study is in line with method [12–16].

## Conclusion

The results show that “pain” is more common among septic patients below the age of 65 and “risk factors for sepsis” is more common among patients below the age of 75. Women with sepsis more often presented with pain and gastrointestinal symptoms than men. However, most keywords related to the presentation of septic patients to the ED had a similar distribution. The results could potentially be used to augment sepsis screening tools or clinical decision tools. Other tools e.g. machine learning may be a method with which to incorporate large amounts of data, including symptoms, to aid in the identification of sepsis.

## Supplementary Information

**Additional file 1.**

## Data Availability

The data used in current study are available from Örebro University, however restrictions do apply to the availability of the data. The data were used under license for the current study and is not publicly available. Upon reasonable request and with the permission from Örebro University the data will be available.

## Abbreviations

ED: Emergency Department
EMS: Emergency Medical Service
HCAI: Healthcare-associated infections
SPSS: Statistical Package of the Social Sciences
IQR: Interquartile range
