# Supplementary material for: Presentation of the septic patient to the emergency department with respect to age and sex – a retrospective cross-sectional study
Source: BMC Emerg Med. 2022 Dec 14;22:205. doi: 10.1186/s12873-022-00759-6 (PMC9749171; doi:10.1186/s12873-022-00759-6)
Supplement: Supplementary file 1 — Additional file 1. [file 12873_2022_759_MOESM1_ESM.docx]

**Supplementary tables**

Supplement to: Larsson E, Wallgren U, Su A, Short J, Kurland L.

**Presentation of the septic patient to the emergency department with respect to age and sex: a retrospective cross-sectional study**

**Table of Contents**

| Supplementary tables | Content | Page |
| --- | --- | --- |
| 1 | Prevalence of primary keywords with respect to age | 2-12 |
| 2 | Prevalence of combined keywords with respect to age | 13-18 |
| 3 | Prevalence of primary keywords with respect to sex | 19-27 |
| 4 | Prevalence of combined keywords with respect to sex | 28-31 |

**Supplementary table 1. Prevalence of primary keywords with respect to age.**

The prevalence of primary keywords* in the entire sample of septic patients presenting to the emergency department of Södersjukhuset during 2013 and prevalence based on age.

|  |  | **Prevalence** | | | | | | | |  |
| --- | --- | --- | --- | --- | --- | --- | --- | --- | --- | --- |
|  |  | **Entire group of ED patients**  **(n=479)** | | **<65 years**  **(n=137)** | | **65-74 years**  **(n=102)** | | **≥75 years**  **(n=240)** | |  |
| **Order** | **Primary keyword *** | **Number of patients** | **Percent (%) and 95% CI** | **Number of patients** | **Percent (%) and 95% CI** | **Number of patients** | **Percent (%) and 95% CI** | **Number of patients** | **Percent (%) and 95% CI** | **P-value**** |
| **1** | **Confirmed fever** Statement fever or statement temperature >38C. | 271 | 56.6  (52.1-61.0) | 85 | 62.0  (53.4-70.2) | 60 | 58.8  (48.6-68.5) | 126 | 52.5  (46.0-59.0) | 0.174 |
| **2** | **Temporal deterioration** Stated deterioration or expressions describing a temporal change | 144 | 30.1  (26.1-34.3) | 43 | 31.4  (23.7-39.9) | 34 | 33.3  (24.3-43.4) | 67 | 27.9  (22.3-34.1) | 0.560 |
| **3** | **Loss of energy** Defined as fatigue, weakness, faintness or similar expressions | 113 | 23.6  (20.0-27.6) | 28 | 20.4  (14.0-28.2) | 19 | 18.6  (11.6-27.6) | 66 | 27.5  (22.0-33.6) | 0.123 |
| **4** | **Low blood pressure** Statement systolic blood pressure ≤90 mmHg | 113 | 23.6  (20.0-27-6) | 33 | 24.1  (17.2-32.1) | 26 | 25.5  (17.4-35.1) | 54 | 22.5  (17.4-28.3) | 0.826 |
| **5** | **Breathing difficulties** Statement difficulties to breath, dyspnea, shortness of breath, shallow breathing or similar expressions | 107 | 22.3  (18.8-26.3) | 22 | 16.1  (10.3-23.3) | 24 | 23.5  (15.7-33.0) | 61 | 25.4  (20.0-31.4) | 0.105 |
| **6** | **Shivering** | 96 | 20.0  (16.7-23.9) | 31 | 22.6  (15.9-30.6) | 27 | 26.5  (18.2-36.1) | 38 | 15.8  (11.5-21.1) | 0.054 |
| **7** | **Decreased general condition** Including expressions such as poor general condition, affected general condition | 94 | 19.6  (16.3-23.4) | 17 | 12.4  (7.4-19.1) | 15 | 14.7  (8.5-23.1) | 62 | 25.8  (20.4-31.9) | 0.003 |
| **8** | **Recent invasive procedures** Including IV drug abuse, surgical and urological procedures, new IV or urinary catheters | 93 | 19.4  (16.1-23.2) | 38 | 27.7  (20.4-36.0) | 25 | 24.5  (16.5-34.0) | 30 | 12.5  (8.6-17.4) | 0.001 |
| **9** | **Abdominal pain** | 92 | 19.2  (15.9-23.0) | 37 | 27.0  (19.8-35.3) | 18 | 17.6  (10.8-26.4) | 37 | 15.4  (11.1-20.6) | 0.021 |
| **10** | **Abnormal behaviour or level of consciousness**  excluding abnormal verbal response | 87 | 18.2  (15.0-21.9) | 17 | 12.4  (7.4-19.1) | 17 | 16.7  (10.0-25.3) | 53 | 22.1  (17.0-27.9) | 0.022 |
| **11** | **Tachypnea** Statement tachypnea, rapid breathing, high respiratory rate, respiratory rate >20 /min, or similar expressions | 84 | 17.5  (14.4-21.2) | 19 | 13.9  (8.6-20.8) | 25 | 24.5  (16.5-34.0) | 40 | 16.7  (12.2-22.0) | 0.089 |
| **12** | **Abnormal verbal response** Defined as no/decreased/changed verbal response | 80 | 16.7  (13.6-20.3) | 19 | 13.9  (8.6-20.8) | 10 | 9.8 (4.8-17.3) | 51 | 21.3  (16.3-27.0) | **0.000109** |
| **13** | **Abnormal micturition**  Defined as hematuria without trauma, bad smelling or cloudy urine, increased frequency of urination | 76 | 15.9  (12.9-19.4) | 14 | 10.2  (5.7-16.6) | 17 | 16.7  (10.0-25.3) | 45 | 18.8  (14.0-24.3) | 0.090 |
| **14** | **Tachycardia** Statement heart rate >90/min or expressions such as rapid heart rate, rapid pulse or similar expressions | 74 | 15.5  (12.5-19.0) | 22 | 16.1  (10.3-23.3) | 18 | 17.6  (10.8-26.4) | 34 | 14.2  (10.0-19.2) | 0.698 |
| **15** | **Reduced intake of food, fluids or oral medicines** Including reduced/no appetite | 73 | 15.2  (12.3-18.7) | 14 | 10.2  (5.7-16.6) | 17 | 16.7  (10.0-25.3) | 42 | 17.5  (12.9-22.9) | 0.151 |
| **16** | **Vomiting** | 71 | 14.8  (11.9-18.3) | 22 | 16.1  (10.3-23.3) | 9 | 8.8 (4.1-16.1) | 40 | 16.7  (12.2-22.0) | 0.156 |
| **17** | **Known ongoing or recent infection** | 66 | 13.8  (11.0-17.2) | 17 | 12.4  (7.4-19.1) | 13 | 12.7  (7.0-20.8) | 36 | 15.0  (10.7-20.2) | 0.737 |
| **18** | **Low oxygen saturation** Defined as statement oxygen saturation <90% (reference clinical praxis for need of oxygen treatment) | 65 | 13.6  (10.8-16.9) | 11 | 8.0 (4.1-13.9) | 14 | 13.7  (7.7-22.0) | 40 | 16.7  (12.2-22.0) | 0.062 |
| **19** | **Cough** | 57 | 11.9  (9.3-15.1) | 19 | 13.9  (8.6-20.8) | 7 | 6.9 (2.8-13.6) | 31 | 12.9  (8.9-17.8) | 0.201 |
| **20** | **Wounds or wound infection** | 55 | 11.5  (8.9-14.7) | 16 | 11.7  (6.8-18.3) | 10 | 9.8 (4.8-17.3) | 29 | 12.1  (8.2-16.9) | 0.830 |
| **21** | **Diarrhoea** | 52 | 10.9  (8.4-14.0) | 21 | 15.3  (9.7-22.5) | 7 | 6.9 (2.8-13.6) | 24 | 10.0  (6.5-14.5) | 0.096 |
| **22** | **Current antibiotic treatment** | 50 | 10.4  (8.0-13.5) | 12 | 8.8 (4.6-14.8) | 13 | 12.7  (7.0-20.8) | 25 | 10.4  (6.9-15.0) | 0.608 |
| **23** | **Airway secretions** Including expectorations, crackles and similar expressions | 48 | 10.0  (7.6-13.0) | 9 | 6.6 (3.0-12.1) | 7 | 6.9 (2.8-13.6) | 32 | 13.3  (9.3-18.3) | 0.053 |
| **24** | **Nausea** | 46 | 9.6  (7.3-12.6) | 22 | 16.1  (10.3-23.3) | 4 | 3.9 (1.1-9.7) | 20 | 8.3 (5.2-12.6) | 0.004 |
| **25** | **Decreased ability to stand or walk**  Including need to carry/lift the patient | 46 | 9.6  (7.3-12.6) | 8 | 5.8 (2.6-11.2) | 9 | 8.8 (4.1-16.1) | 29 | 12.1  (8.2-16.9) | 0.135 |
| **26** | **Oedema/Swelling** | 44 | 9.2  (6.9-12.1) | 20 | 14.6  (9.2-21.6) | 5 | 4.9 (1.6-11.1) | 19 | 7.9 (4.8-12.1) | 0.023 |
| **27** | **Fallen** | 44 | 9.2  (6.9-12.1) | 6 | 4.4  (1.6-9.3) | 10 | 9.8 (4.8-17.3) | 28 | 11.7  (7.9-16.4) | 0.060 |
| **28** | **Substance abuse** Defined as drug abuse, alcohol overconsumption and all other terms indicating substance abuse such as “lives in a home for addicts”, “patient at an outdoor clinic for substance abuse” | 43 | 9.0  (6.7-11.9) | 28 | 20.4  (14.0-28.2) | 12 | 11.8  (6.2-19.6) | 3 | 1.3 (0.3-3.6) | **1.5815E-9** |
| **29** | **Reduced urinary volumes** | 38 | 7.9  (5.8-10.7) | 7 | 5.1  (2.1-10.2) | 11 | 10.8  (5.5-18.5) | 20 | 8.3 (5.2-12.6) | 0.261 |
| **30** | **Extremity pain** | 35 | 7.3  (5.3-10.0) | 17 | 12.4  (7.4-19.1) | 6 | 5.9 (2.2-12.4) | 12 | 5.0 (2.6-8.6) | 0.024 |
| **31** | **Back pain** | 32 | 6.7 (4.8-9.3) | 10 | 7.3 (3.6-13.0) | 9 | 8.8 (4.1-16.1) | 13 | 5.4 (2.9-9.1) | 0.484 |
| **32** | **Suspected fever** Defined as statement feeling hot/warm, increasing temperature or similar expressions | 29 | 6.1 (4.2-8.6) | 12 | 8.8 (4.6-14.8) | 2 | 2.0 (0.2-6.9) | 15 | 6.3 (3.5-10.1) | 0.091 |
| **33** | **Malaise** Defined as expressions such as feeling sick, feeling bad, not feeling well and similar expressions | 27 | 5.6 (3.9-8.1) | 11 | 8.0 (4.1-13.9) | 3 | 2.9 (0.6-8.4) | 13 | 5.4 (2.9-9.1) | 0.236 |
| **34** | **Urinary tract pain** | 27 | 5.6 (3.9-8.1) | 9 | 6.6 (3.0-12.1) | 6 | 5.9 (2.2-12.4) | 12 | 5.0 (2.6-8.6) | 0.811 |
| **35** | **Compromised immune system** Chemotherapy or other immunosuppressive treatment | 26 | 5.4 (3.7-7.8) | 10 | 7.3 (3.6-13.0) | 9 | 8.8 (4.1-16.1) | 7 | 2.9 (1.2-5.9) | 0.046 |
| **36** | **Joint pain** | 25 | 5.2 (3.6-7.6) | 8 | 5.8 (2.6-11.2) | 3 | 2.9 (0.6-8.4) | 14 | 5.8 (3.2-9.6) | 0.507 |
| **37** | **Undefined pain** | 25 | 5.2 (3.6-7.6) | 11 | 8.0 (4.1-13.9) | 4 | 3.9 (1.1-9.7) | 10 | 4.2 (2.0-7.5) | 0.215 |
| **38** | **Chest pain** | 24 | 5.0 (3.4-7.4) | 8 | 5.8 (2.6-11.2) | 3 | 2.9 (0.6-8.4) | 13 | 5.4 (2.9-9.1) | 0.549 |
| **39** | **Focal neurological findings** | 24 | 5.0 (3.4-7.4) | 9 | 6.6 (3.0-12.1) | 5 | 4.9 (1.6-11.1) | 10 | 4.2 (2.0-7.5) | 0.588 |
| **40** | **Found on the floor** or corresponding place | 24 | 5.0 (3.4-7.4) | 4 | 2.9 (0.8-7.3) | 4 | 3.9 (1.1-9.7) | 16 | 6.7 (3.9-10.6) | 0.235 |
| **41** | **General pain** | 23 | 4.8 (3.2-7.1) | 11 | 8.0 (4.1-13.9) | 4 | 3.9 (1.1-9.7) | 8 | 3.3 (1.4-6.5) | 0.133 F* |
| **42** | **Redness (of skin)** | 22 | 4.6 (3.1-6.9) | 9 | 6.6 (3.0-12.1) | 3 | 2.9 (0.6-8.4) | 10 | 4.2 (2.0-7.5) | 0.407 F* |
| **43** | **High CRP** Taken previous to ED arrival | 22 | 4.6 (3.1-6.9) | 9 | 6.6 (3.0-12.1) | 4 | 3.9 (1.1-9.7) | 9 | 3.8 (1.7-7.0) | 0.444 F* |
| **44** | **Reduced amount of stool** | 21 | 4.4 (2.9-6.6) | 3 | 2.2 (0.5-6.3) | 6 | 5.9 (2.2-12.4) | 12 | 5.0 (2.6-8.6) | 0.283 F* |
| **45** | **Positive Pasternatsky´s sign** Costovertebral angle tenderness | 19 | 4.0 (2.6-6.1) | 12 | 8.8 (4.6-14.8) | 1 | 1.0 (0.0-5.3) | 6 | 2.5 (0.9-5.4) | 0.005 F* |
| **46** | **Headache** | 18 | 3.8 (2.4-5.9) | 11 | 8.0 (4.1-13.9) | 4 | 3.9 (1.1-9.7) | 3 | 1.3 (0.3-3.6) | 0.004 F* |
| **47** | **Remained lying or sitting** Statement of being remained sitting or lying in an abnormal way | 18 | 3.8 (2.4-5.9) | 4 | 2.9 (0.8-7.3) | 1 | 1.0 (0.0-5.3) | 13 | 5.4 (2.9-9.1) | 0.118 F* |
| **48** | **Gastrointestinal bleeding** Including melena, hematemesis, hematochezia | 18 | 3.8 (2.4-5.9) | 3 | 2.2 (0.5-6.3) | 4 | 3.9 (1.1-9.7) | 11 | 4.6 (2.3-8.1) | 0.514 F* |
| **49** | **Non-measurable circulatory variables** | 18 | 3.8 (2.4-5.9) | 4 | 2.9 (0.8-7.3) | 6 | 5.9 (2.2-12.4) | 8 | 3.3 (1.4-6.5) | 0.437 F* |
| **50** | **Hypothermia** Defined as statement hypothermia or “very low temp” or statement temperature <36°C | 16 | 3.3 (2.1-5.4) | 2 | 1.5 (0.2-5.2) | 6 | 5.9 (2.2-12.4) | 8 | 3.3 (1.4-6.5) | 0.167 F* |
| **51** | **Irregular pulse** | 16 | 3.3 (2.1-5.4) | 1 | 0.7 (0.0-4.0) | 1 | 1.0 (0.0-5.3) | 14 | 5.8 (3.2-9.6) | 0.010 F* |
| **52** | **High blood sugar** Plasma Glucose >12 mmol/L, regardless diabetes or not. | 14 | 2.9 (1.8-4.8) | 4 | 2.9 (0.8-7.3) | 3 | 2.9 (0.6-8.4) | 7 | 2.9 (1.2-5.9) | 1.000 F* |
| **53** | **Anxiety or fear** | 12 | 2.5 (1.4-4.3) | 4 | 2.9 (0.8-7.3) | 2 | 2.0 (0.2-6.9) | 6 | 2.5 (0.9-5.4) | 1.000 F* |
| **54** | **Fainting-but now awake** | 12 | 2.5 (1.4-4.3) | 5 | 3.6 (1.2-8.3) | 3 | 2.9 (0.6-8.4) | 4 | 1.7 (0.5-4.2) | 0.398 F* |
| **55** | **Sweaty** | 11 | 2.3 (1.3-4.1) | 7 | 5.1 (2.1-10.2) | 1 | 1.0 (0.0-5.4) | 3 | 1.3 (0.3-3.6) | 0.056 F* |
| **56** | **Dysarthria** Slurred speech (but non-affected level of consciousness) | 11 | 2.3 (1.3-4.1) | 3 | 2.2 (0.5-6.3) | 0 | 0.0 (0.0-3.6) | 8 | 3.3 (1.4-6.5) | 0.169 F* |
| **57** | **Dysfunction of urinary catheters** Including obstruction/leakage/problematic urinary catheters including nefrostomias | 10 | 2.1 (1.1-3.8) | 2 | 1.5 (0.2-5.2) | 1 | 1.0 (0.0-5.3) | 7 | 2.9 (1.2-5.9) | 0.529 F* |
| **58** | **Weak pulse or difficulties to palpate the pulse** | 10 | 2.1 (1.1-3.8) | 4 | 2.9 (0.8-7.3) | 4 | 3.9 (1.1-9.7) | 2 | 0.8 (0.1-3.0) | 0.100 F* |
| **59** | **Soiled patient** Patient wetted from his/her own urine or stool | 10 | 2.1 (1.1-3.8) | 4 | 2.9 (0.8-7.3) | 1 | 1.0 (0.0-5.3) | 5 | 2.1 (0.7-4.8) | 0.586 F* |
| **60** | **Obstipation** | 9 | 1.9 (1.0-3.5) | 3 | 2.2 (0.5-6.3) | 5 | 4.9 (1.6-11.1) | 1 | 0.4 (0.0-2.3) | 0.012 F* |
| **61** | **Dizziness** | 9 | 1.9 (1.0-3.5) | 2 | 1.5 (0.2-5.2) | 4 | 3.9 (1.1-9.7) | 3 | 1.3 (0.3-3.6) | 0.296 F* |
| **62** | **Bloodstained patient** | 8 | 1.7 (0.9-3.3) | 5 | 3.6 (1.2-8.3) | 1 | 1.0 (0.0-5.3) | 2 | 0.8 (0.1-3.0) | 0.118 F* |
| **63** | **Peripheral coldness** | 8 | 1.7 (0.9-3.3) | 1 | 0.7 (0.0-4.0) | 4 | 3.9 (1.1-9.7) | 3 | 1.3 (0.3-3.6) | 0.194 F* |
| **64** | **Seizures** | 8 | 1.7 (0.9-3.3) | 4 | 2.9 (0.8-7.3) | 2 | 2.0 (0.2-6.9) | 2 | 0.8 (0.1-3.0) | 0.287 F* |
| **65** | **High blood pressure** Statement high blood pressure or statement blood pressure ≥160 mmHg systolic or ≥100 mmHg diastolic | 7 | 1.5 (0.7-3.0) | 2 | 1.5 (0.2-5.2) | 1 | 1.0 (0.0-5.3) | 4 | 1.7 (0.5-4.2) | 1.000 F* |
| **66** | **Pale** | 7 | 1.5 (0.7-3.0) | 1 | 0.7 (0.0-4.0) | 2 | 2.0 (0.2-6.9) | 4 | 1.7 (0.5-4.2) | 0.695 F* |
| **67** | **History of positive findings in blood culture** Positive blood culture taken previous to EMS arrival-during a visit to the hospital or by other health care provider but the patient is now at home | 6. | 1.3 (0.6-2.7) | 3 | 2.2 (0.5-6.3) | 2 | 2.0 (0.2-6.9) | 1 | 0.4 (0.0-2.3) | 0.179 F* |
| **68** | **Throat pain** | 5 | 1.0 (0.4-2.4) | 3 | 2.2 (0.5-6.3) | 1 | 1.0 (0.0-5.3) | 1 | 0.4 (0.0-2.3) | 0.184 F* |
| **69** | **Mottling** | 5 | 1.0 (0.4-2.4) | 0 | 0.0 (0.0-2.7) | 2 | 2.0 (0.2-6.9) | 3 | 1.3 (0.3-3.6) | 0.297 F* |
| **70** | **Cyanosis** Including blue fingers/nails/lips/toes | 4 | 0.8 (0.3-2.1) | 1 | 0.7 (0.0-4.0) | 0 | 0.0 (0.0-3.6) | 3 | 1.3 (0.3-3.6) | 0.815 F* |
| **71** | **Obstructive breathing** | 4 | 0.8 (0.3-2.1) | 1 | 0.7 (0.0-4.0) | 0 | 0.0 (0.0-3.6) | 3 | 1.3 (0.3-3.6) | 0.815 F* |
| **72** | **Bruises or peteckiae** | 4 | 0.8 (0.3-2.1) | 1 | 0.7 (0.0-4.0) | 1 | 1.0 (0.0-5.3) | 2 | 0.8 (0.1-3.0) | 1.000 F* |
| **73** | **Rash (on skin)** | 3 | 0.6 (0.2-1.8) | 2 | 1.5 (0.2-5.2) | 0 | 0.0 (0.0-3.6) | 1 | 0.4 (0.0-2.3) | 0.314 F* |
| **74** | **Dry mucous membranes (of the mouth)** | 3 | 0.6 (0.2-1.8) | 1 | 0.7 (0.0-4.0) | 0 | 0.0 (0.0-3.6) | 2 | 0.8 (0.1-3.0) | 1.000 F* |
| **75** | **Wound pain** | 2 | 0.4 (0.1-1.5) | 1 | 0.7 (0.0-4.0) | 1 | 1.0 (0.0-5.3) | 0 | 0.0 (0.0-1.5) | 0.248 F* |
| **76** | **Photosensitivity** | 2 | 0.4 (0.1-1.5) | 2 | 1.5 (0.2-5.2) | 0 | 0.0 (0.0-3.6) | 0 | 0.0 (0.0-1.5) | 0.126 F* |
| **77** | **Blisters (on skin)** | 2 | 0.4 (0.1-1.5) | 1 | 0.7 (0.0-4.0) | 1 | 1.0 (0.0-5.3) | 0 | 0.0 (0.0-1.5) | 0.248 F* |
| **78** | **Pale stool** | 2 | 0.4 (0.1-1.5) | 0 | 0.0 (0.0-2.7) | 1 | 1.0 (0.0-5.3) | 1 | 0.4 (0.0-2.3) | 0.462 F* |
| **79** | **Chronically compromised breathing** Such as painful conditions or neurological diseases compromising breathing | 2 | 0.4 (0.1-1.5) | 1 | 0.7 (0.0-4.0) | 0 | 0.0 (0.0-3.6) | 1 | 0.4 (0.0-2.3) | 1.000 F* |
| **80** | **Non-measurable breathing variables** | 2 | 0.4 (0.1-1.5) | 0 | 0.0 (0.0-2.7) | 2 | 2.0 (0.2-6.9) | 0 | 0.0 (0.0-1.5) | 0.045 F* |
| **81** | **Icterus** | 2 | 0.4 (0.1-1.5) | 0 | 0.0 (0.0-2.7) | 2 | 2.0 (0.2-6.9) | 0 | 0.0 (0.0-1.5) | 0.045 F* |
| **82** | **Decreased miscellaneous mobility**  Including expressions such as stiffness when trying to move arms, disability to sit or disability to squeeze the investigators hand | 2 | 0.4 (0.1-1.5) | 0 | 0.0 (0.0-2.7) | 0 | 0.0 (0.0-3.6) | 2 | 0.8 (0.1-3.0) |  |
| **83** | **Cardiac arrest** | 2 | 0.4 (0.1-1.5) | 1 | 0.7 (0.0-4.0) | 0 | 0.0 (0.0-3.6) | 1 | 0.4 (0.0-2.3) | 1.000 F* |
| **84** | **Sensitivity to sound** | 1 | 0.2 (0.0-1.2) | 1 | 0.7 (0.0-4.0) | 0 | 0.0 (0.0-3.6) | 0 | 0.0 (0.0-1.5) | 0.499 F* |
| **85** | **Change of skin turgor** | 1 | 0.2 (0.0-1.2) | 0 | 0.0 (0.0-2.7) | 0 | 0.0 (0.0-3.6) | 1 | 0.4 (0.0-2.3) | 1.000 F* |
| **86** | **Exuding skin** | 1 | 0.2 (0.0-1.2) | 0 | 0.0 (0.0-2.7) | 1 | 1.0 (0.0-5.3) | 0 | 0.0 (0.0-1.5) | 0.213 F* |
| **87** | **Prolonged capillary refill time** | 1 | 0.2 (0.0-1.2) | 0 | 0.0 (0.0-2.7) | 1 | 1.0 (0.0-5.3) | 0 | 0.0 (0.0-1.5) | 0.213 F* |
| **88** | **Painful muscle cramp** | 1 | 0.2 (0.0-1.2) | 1 | 0.7 (0.0-4.0) | 0 | 0.0 (0.0-3.6) | 0 | 0.0 (0.0-1.5) | 0.499 F* |
| **89** | **Palpitations** | 1 | 0.2 (0.0-1.2) | 0 | 0.0 (0.0-2.7) | 0 | 0.0 (0.0-3.6) | 1 | 0.4 (0.0-2.3) | 1.000 F* |
| **90** | **Feeling of depression** | 0 | 0.0 (0.0-0.8) | 0 | 0.0 (0.0-2.7) | 0 | 0.0 (0.0-3.6) | 0 | 0.0 (0.0-1.5) | - |
| EMS= Emergency Medical Services, CI=Confidence Interval, IV= Intravenous, CRP= C-Reactive Protein  * Codes and subcategories derived from the content analysis of septic patients arriving by EMS and admitted to Södersjukhuset during 2012.  **for comparison between the three age groups using chi-square to identify any statistically significant difference. If statistical significance was identified in the first analysis and the total prevalence was >20% a pairwise comparison using chi-square was then performed to identify between which age groups the statistical significance applied to. P-values are presented without adjustment for multiple comparisons. In total 3x90=270 tests were performed. Bonferroni-adjusted significance level is 0,05/270=0,000185. P-values indicating significant differences after adjustment for multiple comparisons by Bonferroni correction are bolded and considered significant in the current study. F* Fischer’s exact test used for statistical analysis due to expected count being <5. | | | | | | | | | | |

**Supplementary table 2. Prevalence of combined keywords with respect to age.**

The prevalence of combined keywords* in the entire sample of septic patients presenting to the emergency department of Södersjukhuset during 2013 and prevalence based on age.

|  |  | **Prevalence** | | | | | | | |  |
| --- | --- | --- | --- | --- | --- | --- | --- | --- | --- | --- |
|  |  | **Entire group of ED patients**  **(n=479)** | | **<65 years**  **(n=137)** | | **65-74 years**  **(n=102)** | | **≥75 years**  **(n=240)** | |  |
| **Order** | **Combined keyword*** | **Number of patients** | **Percent (%) and 95% CI** | **Number of patients** | **Percent (%) and 95% CI** | **Number of patients** | **Percent (%) and 95% CI** | **Number of patients** | **Percent (%) and 95% CI** | **P-value**** |
| **1** | **Abnormal, or suspected abnormal temperature**  In turn including primary keywords shivering OR hypothermia OR the following combined keywords: | 319 | 66.6  (62.3-70.7) | 97 | 70.8  (62.4-78.3) | 72 | 70.6  (60.7-79.2) | 150 | 62.5  (56.0-68.6) | 0.163 |
|  | **-Confirmed or suspected fever** Fever defined as statement fever or statement temperature >38˚C OR suspected fever defined as statement feeling hot/warm, increasing temperature or similar expressions | 296 | 61.8  (57.4-66.0) | 94 | 68.6  (60.1-76.3) | 62 | 60.8  (50.6-70.3) | 140 | 58.3  (51.8-64.6) | 0.138 |
|  | **-Confirmed abnormal temperature (confirmed fever or hypothermia)** Statement fever or statement temperature >38˚C OR statement hypothermia or temperature <36˚C | 286 | 59.7  (55.3-64.0) | 86 | 62.8  (54.1-70.9) | 66 | 64.7  (54.6-73.9) | 134 | 55.8  (49.3-62.2) | 0.213 |
| **2** | **Pain**  Abdominal, extremity, back, undefined, urinary tract, joint, chest, general, headache, throat, wound, painful muscle cramp, positive Pasternatsy´s sign (costovertebral angle tenderness) | 230 | 48.0  (43.6-52.5) | 87 | 63.5  (54.9-71.6) | 44 | 43.1  (33.4-53.3) | 99 | 41.3  (35.0-47.8) | **0.000094** |
| **3** | **Abnormal breathing** Tachypnea, low oxygen saturation, airway secretions, breathing difficulties, cough, or obstructive breathing | 210 | 43.8  (39.5-48.3) | 44 | 32.1  (24.4-40.6) | 51 | 50.0  (39.9-60.1) | 115 | 47.9  (41.4-54.4) | 0.004 |
| **4** | **Risk factors for sepsis** Known ongoing or recent infection, current antibiotic treatment, recent invasive procedures, substance abuse, compromised immune system, chronically compromised breathing | 172 | 36.0  (31.7-40.3) | 74 | 54.0  (45.3-62.6) | 43 | 42.2  (32.4-52.3) | 55 | 22.9  (17.8-28.8) | **3.6677E-9** |
| **5** | **Abnormal circulation** Weak pulse or difficulties to palpate the pulse, peripheral coldness, cardiac arrest, tachycardia, low blood pressure, prolonged capillary refill time or non-measurable circulatory variables | 163 | 34.0  (29.9-38.4) | 47 | 34.3  (26.4-42.9) | 40 | 39.2  (29.7-49.4) | 76 | 31.7  (25.8-38.0) | 0.402 |
| **6** | **Gastrointestinal symptoms** Vomiting, diarrhoea, reduced amount of stool, gastrointestinal bleeding, obstipation, pale faeces | 137 | 28.6  (24.7-32.8) | 44 | 32.1  (24.4-40.6) | 27 | 26.5  (18.2-36.1) | 66 | 27.5  (22.0-33.6) | 0.549 |
| **7** | **Acute altered mental status** Abnormal behaviour or level of consciousness (excluding previously known dementia or mental retardation without statement worse) OR abnormal verbal response defined as no/decreased verbal response | 127 | 26.5  (22.8-30.6) | 28 | 20.4  (14.0-28.2) | 22 | 21.6  (14.0-30.8) | 77 | 32.1  (26.2-38.4) | 0.021 |
| **8** | **Abnormal skin** Pale, wounds or wound infection, sweaty, cyanosis, redness, icterus, mottling, bruises, rash, blisters or peteckiae, change of skin turgor, exuding skin | 125 | 26.1  (22.4-30.2) | 46 | 33.6  (25.7-42.1) | 20 | 19.6  (12.4-28.6) | 59 | 24.6  (19.3-30.5) | 0.039 |
| **9** | **Abnormal urination** Abnormal urination (such as hematuria without trauma, bad smelling or cloudy urine, increased frequency of urination) OR urinary tract pain OR decreased urinary volumes OR dysfunction of urinary catheters defined as obstruction/leakage/problematic urinary catheters including nefrostomias | 118 | 24.6  (21.0-28.7) | 23 | 16.8  (11.0-24.1) | 28 | 27.5  (19.1-37.2) | 67 | 27.9  (22.3-34.1) | 0.041 |
| **10** | **Decreased mobility** In turn including primary keywords remained sitting or lying in an abnormal way OR decreased miscellaneous mobility OR the following combined keywords: | 106 | 22.1  (18.6-26.1) | 18 | 13.1  (8.0-20.0) | 22 | 21.6  (14.0-30.8) | 66 | 27.5  (22.0-33.6) | 0.005 |
|  | **-Weakness of the legs** Decreased ability to stand or walk including need to carry/lift the patient OR fallen OR found on the floor or corresponding place | 93 | 19.4  (16.1-23.2) | 14 | 10.2  (5.7-16.6) | 21 | 20.6  (13.2-29.7) | 58 | 24.2  (18.9-30.1) | 0.004 |
|  | **-Fallen or being found on the floor** or corresponding place | 59 | 12.3  (9.7-15.6) | 8 | 5.8 (2.6-11.2) | 13 | 12.7  (7.0-20.8) | 38 | 15.8  (11.5-21.1) | 0.018 |
| **11** | **History of deranged laboratory tests taken previous to EMS arrival** High blood sugar OR history of high CRP OR history of positive findings in blood culture taken previous to EMS arrival | 43 | 9.0 (6.7-11.9) | 15 | 10.9  (6.3-17.4) | 8 | 7.8  (3.4-14.9) | 20 | 8.3  (5.2-12.6) | 0.627 |
| **12** | **Abnormal neurology** Focal neurological findings, photosensitivity, soundsensetivity, seizures or dysarthria | 40 | 8.4 (6.2-11.2) | 17 | 12.4  (7.4-19.1) | 6 | 5.9  (2.2-12.4) | 17 | 7.1  (4.2-11.1) | 0.119 |
| **13** | **Soiled patient** Bloodstained or wetted from stool or urine | 18 | 3.8 (2.4-5.9) | 9 | 6.6 (3.0-12.1) | 2 | 2.0 (0.2-6.9) | 7 | 2.9 (1.2-5.9) | 0.133 F* |
| **14** | **Mood change** Anxiety or fear OR feeling of depression | 12 | 2.5 (1.4-4.3) | 4 | 2.9 (0.8-7.3) | 2 | 2.0 (0.2-6.9) | 6 | 2.5 (0.9-5.4) | 1.000 F* |
| ED=Emergency Department, EMS= Emergency Medical Services, CI=Confidence Interval, IV= Intravenous  * Consisting of several primary or combined keywords.  **for comparison between the three age groups using chi-square to identify any statistically significant difference. If statistical significance was identified in the first analysis and the total prevalence was >20% a pairwise comparison using chi-square was then performed to identify between which age groups the statistical significance applied to. P-values are presented without adjustment for multiple comparisons. In total 3x17=51 tests were performed. Bonferroni-adjusted significance level is 0,05/36=0,00098. P-values indicating significant differences after adjustment for multiple comparisons by Bonferroni correction are bolded and considered significant in the current study.  F* Fischers exact test used for statistical analysis due to expected count being <5. | | | | | | | | | | |

**Supplementary table 3. Prevalence of primary keywords with respect to sex.**

The prevalence of primary keywords* in the entire sample of septic patients presenting to the emergency department of Södersjukhuset during 2013 and prevalence based on sex.

|  |  | **Prevalence** | | | | | |  |
| --- | --- | --- | --- | --- | --- | --- | --- | --- |
|  |  | **Entire group of ED patients**  **(n=479)** | | **Women**  **(n=224)** | | **Men**  **(n=255)** | |  |
| **Order** | **Primary keyword*** | **Number of patients** | **Percent (%) and 95% CI** | **Number of patients** | **Percent (%) and 95% CI** | **Number of patients** | **Percent (%) and 95% CI** | **P-value**** |
| **1** | **Confirmed fever** Statement fever or statement temperature >38˚C | 271 | 56.6 (52.1-61.0) | 132 | 58.9 (52.2-65.4) | 139 | 54.5 (48.2-60.7) | 0.330 |
| **2** | **Temporal deterioration** Stated deterioration or expressions describing a temporal change | 144 | 30.1 (26.1-34.3) | 69 | 30.8 (24.8-37.3) | 75 | 29.4 (23.9-35.4) | 0.740 |
| **3** | **Loss of energy** Defined as fatigue, weakness, faintness or similar expressions | 113 | 23.6 (20.0-27.6) | 59 | 26.3 (20.7-32.6) | 54 | 21.2 (16.3-26.7) | 0.184 |
| **4** | **Low blood pressure** Statement systolic blood pressure ≤90 mmHg | 113 | 23.6 (20.0-27-6) | 60 | 26.8 (21.1-33.1) | 53 | 20.8 (16.0-26.3) | 0.123 |
| **5** | **Breathing difficulties** Statement difficulties to breath, dyspnea, shortness of breath, shallow breathing or similar expressions | 107 | 22.3 (18.8-26.3) | 50 | 22.3 (17.0-28.3) | 57 | 22.4 (17.4-28.0) | 0.993 |
| **6** | **Shivering** | 96 | 20.0 (16.7-23.9) | 44 | 19.6 (14.7-25.5) | 52 | 20.4 (15.6-25.9) | 0.838 |
| **7** | **Decreased general condition** Including expressions such as poor general condition, affected general condition | 94 | 19.6 (16.3-23.4) | 56 | 25.0 (19.5-31.2) | 38 | 14.9 (10.8-19.9) | 0.005 |
| **8** | **Recent invasive procedures** Including IV drug abuse, surgical and urological procedures, new IV or urinary catheters | 93 | 19.4 (16.1-23.2) | 34 | 15.2 (10.7-20.6) | 59 | 23.1 (18.1-28.8) | 0.028 |
| **9** | **Abdominal pain** | 92 | 19.2 (15.9-23.0) | 57 | 25.4 (19.9-31.7) | 35 | 13.7 (9.8-18.6) | 0.001 |
| **10** | **Abnormal behaviour or level of consciousness**  excluding abnormal verbal response | 87 | 18.2 (15.0-21.9) | 37 | 16.5 (11.9-22.0) | 50 | 19.6 (14.9-25.0) | 0.673 |
| **11** | **Tachypnea** Statement tachypnea, rapid breathing, high respiratory rate, respiratory rate >20/min, or similar expressions | 84 | 17.5 (14.4-21.2) | 37 | 16.5 (11.9-22.0) | 47 | 18.4 (13.9-23.7) | 0.583 |
| **12** | **Abnormal verbal response** Defined as no/decreased/changed verbal response | 80 | 16.7 (13.6-20.3) | 39 | 17.4 (12.7-23.0) | 41 | 16.1 (11.8-21.2) | 0.467 |
| **13** | **Abnormal micturition** Defined as hematuria without trauma, bad smelling or cloudy urine, increased frequency of urination | 76 | 15.9 (12.9-19.4) | 27 | 12.1 (8.1-17.1) | 49 | 19.2 (14.6-24.6) | 0.032 |
| **14** | **Tachycardia** Statement heart rate >90/min or expressions such as rapid heart rate, rapid pulse or similar expressions | 74 | 15.5 (12.5-19.0) | 35 | 15.6 (11.1-21.1) | 39 | 15.3 (11.1-20.3) | 0.920 |
| **15** | **Reduced intake of food, fluids or oral medicines** Including reduced/no appetite | 73 | 15.2 (12.3-18.7) | 48 | 21.4 (16.2-27.4) | 25 | 9.8 (6.4-14.1) | 0.000412 |
| **16** | **Vomiting** | 71 | 14.8 (11.9-18.3) | 52 | 23.2 (17.9-29.3) | 19 | 7.5 (4.5-11.4) | **0.000001** |
| **17** | **Known ongoing or recent infection** | 66 | 13.8 (11.0-17.2) | 32 | 14.3 (10.0-19.6) | 34 | 13.3 (9.4-18.1) | 0.763 |
| **18** | **Low oxygen saturation** Defined as statement oxygen saturation <90% (reference clinical praxis for need of oxygen treatment) | 65 | 13.6 (10.8-16.9) | 32 | 14.3 (10.0-19.6) | 33 | 12.9 (9.1-17.7) | 0.668 |
| **19** | **Cough** | 57 | 11.9 (9.3-15.1) | 33 | 14.7 (10.4-20.1) | 24 | 9.4 (6.1-13.7) | 0.073 |
| **20** | **Wounds or wound infection** | 55 | 11.5 (8.9-14.7) | 24 | 10.7 (7.0-15.5) | 31 | 12.2 (8.4-16.8) | 0.621 |
| **21** | **Diarrhoea** | 52 | 10.9 (8.4-14.0) | 34 | 15.2 (10.7-20.6) | 18 | 7.1 (4.2-10.9) | 0.004 |
| **22** | **Current antibiotic treatment** | 50 | 10.4 (8.0-13.5) | 17 | 7.6 (4.5-11.9) | 33 | 12.9 (9.1-17.7) | 0.056 |
| **23** | **Airway secretions** Including expectorations, crackles and similar expressions | 48 | 10.0 (7.6-13.0) | 27 | 12.1 (8.1-17.1) | 21 | 8.2 (5.2-12.3) | 0.165 |
| **24** | **Nausea** | 46 | 9.6 (7.3-12.6) | 33 | 14.7 (10.4-20.1) | 13 | 5.1 (2.7-8.6) | 0.000356 |
| **25** | **Decreased ability to stand or walk**  Including need to carry/lift the patient | 46 | 9.6 (7.3-12.6) | 16 | 7.1 (4.1-11.3) | 30 | 11.8 (8.1-16.4) | 0.087 |
| **26** | **Oedema/Swelling** | 44 | 9.2 (6.9-12.1) | 20 | 8.9 (5.5-13.5) | 24 | 9.4 (6.1-13.7) | 0.855 |
| **27** | **Fallen** | 44 | 9.2 (6.9-12.1) | 15 | 6.7 (3.8-10.8) | 29 | 11.4 (7.8-15.9) | 0.077 |
| **28** | **Substance abuse** Defined as drug abuse, alcohol overconsumption and all other terms indicating substance abuse such as “lives in a home for addicts”, “patient at an outdoor clinic for substance abuse” | 43 | 9.0 (6.7-11.9) | 17 | 7.6 (4.5-11.9) | 26 | 10.2 (6.8-14.6) | 0.319 |
| **29** | **Reduced urinary volumes** | 38 | 7.9 (5.8-10.7) | 16 | 7.1 (4.1-11.3) | 22 | 8.6 (5.5-12.8) | 0.549 |
| **30** | **Extremity pain** | 35 | 7.3 (5.3-10.0) | 23 | 10.3 (6.6-15.0) | 12 | 4.7 (2.5-8.1) | 0.020 |
| **31** | **Back pain** | 32 | 6.7 (4.8-9.3) | 19 | 8.5 (5.2-12.9) | 13 | 5.1 (2.7-8.6) | 0.139 |
| **32** | **Suspected fever** Defined as statement feeling hot/warm, increasing temperature or similar expressions | 29 | 6.1 (4.2-8.6) | 15 | 6.7 (3.8-10.8) | 14 | 5.5 (3.0-9.0) | 0.581 |
| **33** | **Malaise** Defined as expressions such as feeling sick, feeling bad, not feeling well and similar expressions | 27 | 5.6 (3.9-8.1) | 13 | 5.8 (3.1-9.7) | 14 | 5.5 (3.0-9.0) | 0.882 |
| **34** | **Urinary tract pain** | 27 | 5.6 (3.9-8.1) | 12 | 5.4 (2.8-9.2) | 15 | 5.9 (3.3-9.5) | 0.804 |
| **35** | **Compromised immune system** Chemotherapy or other immunosuppressive treatment | 26 | 5.4 (3.7-7.8) | 13 | 5.8 (3.1-9.7) | 13 | 5.1 (2.7-8.6) | 0.734 |
| **36** | **Joint pain** | 25 | 5.2 (3.6-7.6) | 10 | 4.5 (2.2-8.1) | 15 | 5.9 (3.3-9.5) | 0.486 |
| **37** | **Undefined pain** | 25 | 5.2 (3.6-7.6) | 13 | 5.8 (3.1-9.7) | 12 | 4.7 (2.5-8.1) | 0.590 |
| **38** | **Chest pain** | 24 | 5.0 (3.4-7.4) | 16 | 7.1 (4.1-11.3) | 8 | 3.1 (1.4-6.1) | 0.045 |
| **39** | **Focal neurological findings** | 24 | 5.0 (3.4-7.4) | 10 | 4.5 (2.2-8.1) | 14 | 5.5 (3.0-9.0) | 0.608 |
| **40** | **Found on the floor** or corresponding place | 24 | 5.0 (3.4-7.4) | 14 | 6.3 (3.5-10.3) | 10 | 3.9 (1.9-7.1) | 0.244 |
| **41** | **General pain** | 23 | 4.8 (3.2-7.1) | 12 | 5.4 (2.8-9.2) | 11 | 4.3 (2.2-7.6) | 0.594 |
| **42** | **Redness (of skin)** | 22 | 4.6 (3.1-6.9) | 10 | 4.5 (2.2-8.1) | 12 | 4.7 (2.5-8.1) | 0.900 |
| **43** | **High CRP** Taken previous to ED arrival | 22 | 4.6 (3.1-6.9) | 11 | 4.9 (2.6-8.6) | 11 | 4.3 (2.2-7.6) | 0.755 |
| **44** | **Reduced amount of stool** | 21 | 4.4 (2.9-6.6) | 13 | 5.8 (3.1-9.7) | 8 | 3.1 (1.4-6.1) | 0.155 |
| **45** | **Positive Pasternatsky´s sign** Costovertebral angle tenderness | 19 | 4.0 (2.6-6.1) | 13 | 5.8 (3.1-9.7) | 6 | 2.4 (0.9-5.1) | 0.054 |
| **46** | **Headache** | 18 | 3.8 (2.4-5.9) | 9 | 4.0 (1.9-7.5) | 9 | 3.5 (1.6-6.6) | 0.779 |
| **47** | **Remained lying or sitting** Statement of being remained sitting or lying in an abnormal way | 18 | 3.8 (2.4-5.9) | 11 | 4.9 (2.6-8.6) | 7 | 2.7 (1.1-5.6) | 0.214 |
| **48** | **Gastrointestinal bleeding** Including melena, hematemesis, hematochezia | 18 | 3.8 (2.4-5.9) | 5 | 2.2 (0.7-5.1) | 13 | 5.1 (2.7-8.6) | 0.100 |
| **49** | **Non-measurable circulatory variables** | 18 | 3.8 (2.4-5.9) | 9 | 4.0 (1.9-7.5) | 9 | 3.5 (1.6-6.6) | 0.779 |
| **50** | **Hypothermia** Defined as statement hypothermia or “very low temp” or statement temperature <36°C | 16 | 3.3 (2.1-5.4) | 5 | 2.2 (0.7-5.1) | 11 | 4.3 (2.2-7.6) | 0.206 |
| **51** | **Irregular pulse** | 16 | 3.3 (2.1-5.4) | 8 | 3.6 (1.6-6.9) | 8 | 3.1 (1.4-6.1) | 0.792 |
| **52** | **High blood sugar** Plasma Glucose >12 mmol/L, regardless diabetes or not. | 14 | 2.9 (1.8-4.8) | 5 | 2.2 (0.7-5.1) | 9 | 3.5 (1.6-6.6) | 0.400 |
| **53** | **Anxiety or fear** | 12 | 2.5 (1.4-4.3) | 4 | 1.8 (0.5-4.5) | 8 | 3.1 (1.4-6.1) | 0.345 |
| **54** | **Fainting-but now awake** | 12 | 2.5 (1.4-4.3) | 6 | 2.7 (1.0-5.7) | 6 | 2.4 (0.9-5.1) | 0.820 |
| **55** | **Sweaty** | 11 | 2.3 (1.3-4.1) | 4 | 1.8 (0.5-4.5) | 7 | 2.7 (1.1-5.6) | 0.484 |
| **56** | **Dysarthria** Slurred speech (but non-affected level of consciousness) | 11 | 2.3 (1.3-4.1) | 7 | 3.1 (1.3-6.3) | 4 | 1.6 (0.4-4.0) | 0.257 |
| **57** | **Dysfunction of urinary catheters** Including obstruction/leakage/problematic urinary catheters including nefrostomias | 10 | 2.1 (1.1-3.8) | 0 | 0.0 (0.0-1.6) | 10 | 3.9 (1.9-7.1) | 0.002 F* |
| **58** | **Weak pulse or difficulties to palpate the pulse** | 10 | 2.1 (1.1-3.8) | 3 | 1.3 (0.3-3.9) | 7 | 2.7 (1.1-5.6) | 0.349 F* |
| **59** | **Soiled patient** Patient wetted from his/her own urine or stool | 10 | 2.1 (1.1-3.8) | 6 | 2.7 (1.0-5.7) | 4 | 1.6 (0.4-4.0) | 0.526 F* |
| **60** | **Obstipation** | 9 | 1.9 (1.0-3.5) | 3 | 1.3 (0.3-3.9) | 6 | 2.4 (0.9-5.1) | 0.512 F* |
| **61** | **Dizziness** | 9 | 1.9 (1.0-3.5) | 3 | 1.3 (0.3-3.9) | 6 | 2.4 (0.9-5.1) | 0.512 F* |
| **62** | **Bloodstained patient** | 8 | 1.7 (0.9-3.3) | 7 | 3.1 (1.3-6.3) | 1 | 0.4 (0.0-2.2) | 0.028 F* |
| **63** | **Peripheral coldness** | 8 | 1.7 (0.9-3.3) | 4 | 1.8 (0.5-4.5) | 4 | 1.6 (0.4-4.0) | 1.000 F* |
| **64** | **Seizures** | 8 | 1.7 (0.9-3.3) | 4 | 1.8 (0.5-4.5) | 4 | 1.6 (0.4-4.0) | 1.000 F* |
| **65** | **High blood pressure** Statement high blood pressure or statement blood pressure ≥160 mmHg systolic or ≥100 mmHg diastolic | 7 | 1.5 (0.7-3.0) | 3 | 1.3 (0.3-3.9) | 4 | 1.6 (0.4-4.0) | 1.000 F* |
| **66** | **Pale** | 7 | 1.5 (0.7-3.0) | 3 | 1.3 (0.3-3.9) | 4 | 1.6 (0.4-4.0) | 1.000 F* |
| **67** | **History of positive findings in blood culture** Positive blood culture taken previous to EMS arrival-during a visit to the hospital or by other health care provider but the patient is now at home | 6 | 1.3 (0.6-2.7) | 3 | 1.3 (0.3-3.9) | 3 | 1.2 (0.2-3.4) | 1.000 F* |
| **68** | **Throat pain** | 5 | 1.0 (0.4-2.4) | 4 | 1.8 (0.5-4.5) | 1 | 0.4 (0.0-2.2) | 0.190 F* |
| **69** | **Mottling** | 5 | 1.0 (0.4-2.4) | 2 | 0.9 (0.1-3.2) | 3 | 1.2 (0.2-3.4) | 1.000 F* |
| **70** | **Cyanosis** Including blue fingers/nails/lips/toes | 4 | 0.8 (0.3-2.1) | 1 | 0.4 (0.0-2.5) | 3 | 1.2 (0.2-3.4) | 0.627 F* |
| **71** | **Obstructive breathing** | 4 | 0.8 (0.3-2.1) | 1 | 0.4 (0.0-2.5) | 3 | 1.2 (0.2-3.4) | 0.627 F* |
| **72** | **Bruises or peteckiae** | 4 | 0.8 (0.3-2.1) | 2 | 0.9 (0.1-3.2) | 2 | 0.8 (0.1-2.8) | 1.000 F* |
| **73** | **Rash (on skin)** | 3 | 0.6 (0.2-1.8) | 3 | 1.3 (0.3-3.9) | 0 | 0.0 (0.0-1.4) | 0.102 F* |
| **74** | **Dry mucous membranes (of the mouth)** | 3 | 0.6 (0.2-1.8) | 3 | 1.3 (0.3-3.9) | 0 | 0.0 (0.0-1.4) | 0.102 F* |
| **75** | **Wound pain** | 2 | 0.4 (0.1-1.5) | 1 | 0.4 (0.0-2.5) | 1 | 0.4 (0.0-2.2) | 1.000 F* |
| **76** | **Photosensitivity** | 2 | 0.4 (0.1-1.5) | 2 | 0.9 (0.1-3.2) | 0 | 0.0 (0.0-1.4) | 0.218 F* |
| **77** | **Blisters (on skin)** | 2 | 0.4 (0.1-1.5) | 2 | 0.9 (0.1-3.2) | 0 | 0.0 (0.0-1.4) | 0.218 F* |
| **78** | **Pale stool** | 2 | 0.4 (0.1-1.5) | 2 | 0.9 (0.1-3.2) | 0 | 0.0 (0.0-1.4) | 0.218 F* |
| **79** | **Chronically compromised breathing** Such as painful conditions or neurological diseases compromising breathing | 2 | 0.4 (0.1-1.5) | 1 | 0.4 (0.0-2.5) | 1 | 0.4 (0.0-2.2) | 1.000 F* |
| **80** | **Non-measurable breathing variables** | 2 | 0.4 (0.1-1.5) | 0 | 0.0 (0.0-1.6) | 2 | 0.8 (0.1-2.8) | 0.501 F* |
| **81** | **Icterus** | 2 | 0.4 (0.1-1.5) | 0 | 0.0 (0.0-1.6) | 2 | 0.8 (0.1-2.8) | 0.501 F* |
| **82** | **Decreased miscellaneous mobility**  Including expressions such as stiffness when trying to move arms, disability to sit or disability to squeeze the investigators hand | 2 | 0.4 (0.1-1.5) | 0 | 0.0 (0.0-1.6) | 2 | 0.8 (0.1-2.8) | 0.501 F* |
| **83** | **Cardiac arrest** | 2 | 0.4 (0.1-1.5) | 0 | 0.0 (0.0-1.6) | 2 | 0.8 (0.1-2.8) | 0.501 F* |
| **84** | **Sensitivity to sound** | 1 | 0.2 (0.0-1.2) | 0 | 0.0 (0.0-1.6) | 1 | 0.4 (0.0-2.2) | 1.000 F* |
| **85** | **Change of skin turgor** | 1 | 0.2 (0.0-1.2) | 0 | 0.0 (0.0-1.6) | 1 | 0.4 (0.0-2.2) | 1.000 F* |
| **86** | **Exuding skin** | 1 | 0.2 (0.0-1.2) | 0 | 0.0 (0.0-1.6) | 1 | 0.4 (0.0-2.2) | 1.000 F* |
| **87** | **Prolonged capillary refill time** | 1 | 0.2 (0.0-1.2) | 0 | 0.0 (0.0-1.6) | 1 | 0.4 (0.0-2.2) | 1.000 F* |
| **88** | **Painful muscle cramp** | 1 | 0.2 (0.0-1.2) | 1 | 0.4 (0.0-2.5) | 0 | 0.0 (0.0-1.4) | 0.468 F* |
| **89** | **Palpitations** | 1 | 0.2 (0.0-1.2) | 1 | 0.4 (0.0-2.5) | 0 | 0.0 (0.0-1.4) | 0.468 F* |
| **90** | **Feeling of depression** | 0 | 0.0 (0.0-0.8) | 0 | 0.0 (0.0-1.6) | 0 | 0.0 (0.0-1.4) | - |
| ED=Emergency Department, CI=Confidence Interval, IV= Intravenous, CRP= C-Reactive Protein  *Codes and subcategories derived from the content analysis of septic patients arriving by EMS and admitted to Södersjukhuset during 2012.  **for comparison between sexes. P-values are presented without adjustment for multiple comparisons. In total 2x90=180 tests were performed. Bonferroni-adjusted significance level is 0,05/180=0,00028. P-values indicating significant differences after adjustment for multiple comparisons by Bonferroni correction are bolded and considered significant in the current study. F* Fischer’s exact test used for statistical analysis due to expected count being <5. | | | | | | | | |

**Supplementary table 4. Prevalence of combined keywords with respect to sex.**

The prevalence of combined keywords* in the entire sample of septic patients presenting to the emergency department of Södersjukhuset during 2013 and prevalence based on sex.

|  |  | **Prevalence** | | | | | |  |
| --- | --- | --- | --- | --- | --- | --- | --- | --- |
|  |  | **Entire group of ED patients**  **(n=479)** | | **Women**  **(n=224)** | | **Men**  **(n=255)** | |  |
| **Order** | **Combined keyword*** | **Number of patients** | **Percent (%) and 95% CI** | **Number of patients** | **Percent (%) and 95% CI** | **Number of patients** | **Percent (%) and 95% CI** | **P-value**** |
| **1** | **Abnormal, or suspected abnormal temperature**  In turn including primary keywords shivering OR hypothermia OR the following combined keywords: | 319 | 66.6 (62.3-70.7) | 155 | 69.2 (62.7-75.2) | 164 | 64.3 (58.1-70.2) | 0.258 |
|  | **-Confirmed or suspected fever** Fever defined as statement fever or statement temperature >38˚C OR suspected fever defined as statement feeling hot/warm, increasing temperature or similar expressions | 296 | 61.8 (57.4-66.0) | 145 | 64.7 (58.1-71.0) | 151 | 59.2 (52.9-65.3) | 0.215 |
|  | **-Confirmed abnormal temperature (confirmed fever or hypothermia)** Statement fever or statement temperature >38˚[1] OR statement hypothermia or temperature <36˚C | 286 | 59.7 (55.3-64.0) | 137 | 61.2 (54.4-67.6) | 149 | 58.4 (52.1-64.5) | 0.543 |
| **2** | **Pain**  Abdominal, extremity, back, undefined, urinary tract, joint, chest, general, headache, throat, wound, painful muscle cramp, positive Pasternatsy´s sign (costovertebral angle tenderness) | 230 | 48.0 (43.6-52.5) | 128 | 57.1 (50.4-63.7) | 102 | 40.0 (33.9-46.3) | **0.000179** |
| **3** | **Abnormal breathing** Tachypnea, low oxygen saturation, airway secretions, breathing difficulties, cough, or obstructive breathing | 210 | 43.8 (39.5-48.3) | 104 | 46.4 (39.8-53.2) | 106 | 41.6 (35.5-47.9) | 0.285 |
| **4** | **Risk factors for sepsis** Known ongoing or recent infection, current antibiotic treatment, recent invasive procedures, substance abuse, compromised immune system, chronically compromised breathing | 172 | 36.0 (31.7-40.3) | 72 | 32.1 (26.1-38.7) | 100 | 39.2 (33.2-45.5) | 0.107 |
| **5** | **Abnormal circulation** Weak pulse or difficulties to palpate the pulse, peripheral coldness, cardiac arrest, tachycardia, low blood pressure, prolonged capillary refill time or non-measurable circulatory variables | 163 | 34.0 (29.9-38.4) | 83 | 37.1 (30.7-43.7) | 80 | 31.4 (25.7-37.5) | 0.190 |
| **6** | **Gastrointestinal symptoms** Vomiting, diarrhoea, reduced amount of stool, gastrointestinal bleeding, obstipation, pale faeces | 137 | 28.6 (24.7-32.8) | 82 | 36.6 (30.3-43.3) | 55 | 21.6 (16.7-27.1) | **0.000279** |
| **7** | **Acute altered mental status** Abnormal behaviour or level of consciousness (excluding previously known dementia or mental retardation without statement worse) OR abnormal verbal response defined as no/decreased verbal response | 127 | 26.5 (22.8-30.6) | 58 | 25.9 (20.3-32.1) | 69 | 27.1 (21.7-33.0) | 0.773 |
| **8** | **Abnormal skin** Pale, wounds or wound infection, sweaty, cyanosis, redness, icterus, mottling, bruises, rash, blisters or peteckiae, change of skin turgor, exuding skin | 125 | 26.1 (22.4-30.2) | 59 | 26.3 (20.7-32.6) | 66 | 25.9 (20.6-31.7) | 0.910 |
| **9** | **Abnormal urination** Abnormal urination (such as hematuria without trauma, bad smelling or cloudy urine, increased frequency of urination) OR urinary tract pain OR decreased urinary volumes OR dysfunction of urinary catheters defined as obstruction/leakage/problematic urinary catheters including nefrostomias | 118 | 24.6 (21.0-28.7) | 46 | 20.5 (15.4-26.4) | 72 | 28.2 (22.8-34.2) | 0.051 |
| **10** | **Decreased mobility** In turn including primary keywords remained sitting or lying in an abnormal way OR decreased miscellaneous mobility OR the following combined keywords: | 106 | 22.1 (18.6-26.1) | 41 | 18.3 (13.5-24.0) | 65 | 25.5 (20.3-31.3) | 0.059 |
|  | **-Weakness of the legs** Decreased ability to stand or walk including need to carry/lift the patient OR fallen OR found on the floor or corresponding place | 93 | 19.4 (16.1-23.2) | 33 | 14.7 (10.4-20.1) | 60 | 23.5 (18.5-29.2) | 0.015 |
|  | **-Fallen or being found on the floor** or corresponding place | 59 | 12.3 (9.7-15.6) | 24 | 10.7 (7.0-15.5) | 35 | 13.7 (9.8-18.6) | 0.317 |
| **11** | **History of deranged laboratory tests taken previous to EMS arrival** High blood sugar OR history of high CRP OR history of positive findings in blood culture taken previous to EMS arrival | 43 | 9.0 (6.7-11.9) | 18 | 8.0 (4.8-12.4) | 25 | 9.8 (6.4-14.1) | 0.499 |
| **12** | **Abnormal neurology** Focal neurological findings, photosensitivity, soundsensetivity, seizures or dysarthria | 40 | 8.4 (6.2-11.2) | 20 | 8.9 (5.5-13.5) | 20 | 7.8 (4.9-11.9) | 0.668 |
| **13** | **Soiled patient** Bloodstained or wetted from stool or urine | 18 | 3.8 (2.4-5.9) | 13 | 5.8 (3.1-9.7) | 5 | 2.0 (0.6-4.5) | 0.027 |
| **14** | **Mood change** Anxiety or fear OR feeling of depression | 12 | 2.5 (1.4-4.3) | 4 | 1.8 (0.5-4.5) | 8 | 3.1 (1.4-6.1) | 0.345 |
| ED=Emergency Department, CI=Confidence Interval, IV= Intravenous, CRP= C-Reactive Protein, EMS=Emergency Medical Services.  * Consisting of several primary or combined keywords.  ****for comparison between sexes. P-values are presented without adjustment for multiple comparisons. In total 2x18=36 tests were performed. Bonferroni-adjusted significance level is 0,05/36=0,00139. P-values indicating significant differences after adjustment for multiple comparisons by Bonferroni correction are bolded and considered significant in the current study. F* Fischers exact test used for statistical analysis due to expected count being <5. | | | | | | | | |
